# Alcohol and Drug Use Disorders among Patients with Myocardial Infarction: Associations with Disparities in Care and Mortality

**DOI:** 10.1371/journal.pone.0066551

**Published:** 2013-09-11

**Authors:** Cynthia A. Beck, Danielle A. Southern, Richard Saitz, Merril L. Knudtson, William A. Ghali

**Affiliations:** 1 Department of Community Health Sciences, University of Calgary, Calgary, Alberta, Canada; 2 Department of Psychiatry, University of Calgary, Calgary, Alberta, Canada; 3 Clinical Addiction Research and Education Unit, Section of General Internal Medicine, Department of Medicine, Boston Medical Center and Boston University School of Medicine, Boston, Massachusetts, United States of America; 4 Department of Epidemiology, Boston University School of Public Health, Boston, Massachusetts, United States of America; 5 Faculty of Medicine, University of Calgary, Calgary, Alberta, Canada; 6 Institute for Public Health (IPH), University of Calgary, Calgary, Alberta, Canada; Sapienza University of Rome, Italy

## Abstract

**Background:**

Because alcohol and drug use disorders (SUDs) can influence quality of care, we compared patients with and without SUDs on frequency of catheterization, revascularization, and in-hospital mortality after acute myocardial infarction (AMI).

**Methods:**

This study employed hospital discharge data identifying all adult AMI admissions (ICD-9-CM code 410) between April 1996 and December 2001. Patients were classified as having an SUD if they had alcohol and/or drug (not nicotine) abuse or dependence using a validated ICD-9-CM coding definition. Catheterization and revascularization data were obtained by linkage with a clinically-detailed cardiac registry. Analyses (controlling for comorbidities and disease severity) compared patients with and without SUDs for post-MI catheterization, revascularization, and in-hospital mortality.

**Results:**

Of 7,876 AMI unique patient admissions, 2.6% had an SUD. In adjusted analyses mortality was significantly higher among those with an SUD (odds ratio (OR) 2.02; 95%CI: 1.10–3.69), while there was a trend toward lower catheterization rates among those with an SUD (OR 0.75; 95%CI: 0.55–1.01). Among the subset of AMI admissions who underwent catheterization, the adjusted hazard ratio for one-year revascularization was 0.85 (95%CI: 0.65–1.11) with an SUD compared to without.

**Conclusions:**

Alcohol and drug use disorders are associated with significantly higher in-hospital mortality following AMI in adults of all ages, and may also be associated with decreased access to catheterization and revascularization. This higher mortality in the face of poorer access to procedures suggests that these individuals may be under-treated following AMI. Targeted efforts are required to explore the interplay of patient and provider factors that underlie this finding.

## Introduction

Alcohol and other drug use disorders (ie. substance use disorders, SUDs) can influence outcomes of other health conditions through a variety of mechanisms, including suboptimal quality of care. It is well known that physicians vary in their decision-making regarding the appropriateness of procedures [1]–[3]. Both clinical and non-clinical factors seem to enter into these decisions, and certain sociodemographic and insurance groups have been shown to receive fewer cardiovascular procedures than would be expected given their clinical condition [4]–[9]. Patient preferences and behaviors may also play a role [10]. Moreover, individuals with SUDs have been shown to have decreased access to medical and preventive health care [11]–[13].

There have been several studies that have shown the superiority of percutaneous coronary intervention (PCI) as a treatment for ST-elevation myocardial infarction (STEMI) when compared with thrombolytic therapy in terms of reducing mortality rate and recurrence of myocardial infarction (MI) [14]–[16]. Prior studies have reported discrepant results on the association between substance use disorders and access to these cardiovascular procedures following acute myocardial infarct (AMI) [11]. Druss and colleagues showed that those with SUDs aged 65 and older in acute care non-governmental hospitals in the United States were less likely than those with neither mental nor substance use disorder to receive cardiac catheterization after AMI [17]. Young reported data from a large all-payer American database stratified by age, indicating that patients with substance use disorders both younger and older than 65 years have lower rates of cardiac procedures and higher rates of in-hospital mortality compared with those with neither mental nor substance use disorders [18]. However, this study did not adjust for admission characteristics or left ventricular function. Most recently, Jones and colleagues published results from an analysis of administrative data from a large commercially insured sample of all ages, adjusting for comorbidities [19]. They obtained an unexpected result: those with a substance use disorder were more likely to receive percutaneous coronary intervention (PCI) compared to those with neither mental nor substance use disorders. This finding did not carry over to coronary artery bypass grafting (CABG). Li and colleagues looked at a related issue and demonstrated that in New York hospitals, individuals with substance use plus mental disorders but not substance use disorders alone were more likely to have their CABG surgery performed by low quality surgeons [20].

As such, it is as yet unclear whether having a known substance use disorder affects receipt of cardiovascular procedures, and the only study for which analyses were adjusted for clinical characteristics such as ejection fraction used a sample over 65 years of age [17]. The availability of both hospital discharge data and the Alberta Provincial Project for Outcome Assessment in Coronary Heart Disease (APPROACH: www.approach.org), a linkable population-based registry of cardiovascular procedures, presented an opportunity to study this issue in Alberta, Canada [21]. These Canadian data had the additional advantage that any disparities detected in the analysis would be unlikely to be caused by lack of health insurance among patients with SUDs. To our knowledge there are no published Canadian studies to date on this issue. A further advantage of our data was that they contained information on patients' discharge status, allowing examination of the effect of discharge against medical advice (AMA) on the results. Discharge AMA has been shown to affect relationships between service utilization and substance use, and many studies have failed to take this issue into account [22], [23].

Our objective was to study AMI patients in APPROACH to compare the in-hospital mortality and the proportions receiving catheterization and revascularization, between those with and without a known alcohol or drug use disorder.

## Methods

### Data Sources, Subjects, and Variables

**Figure 1:**
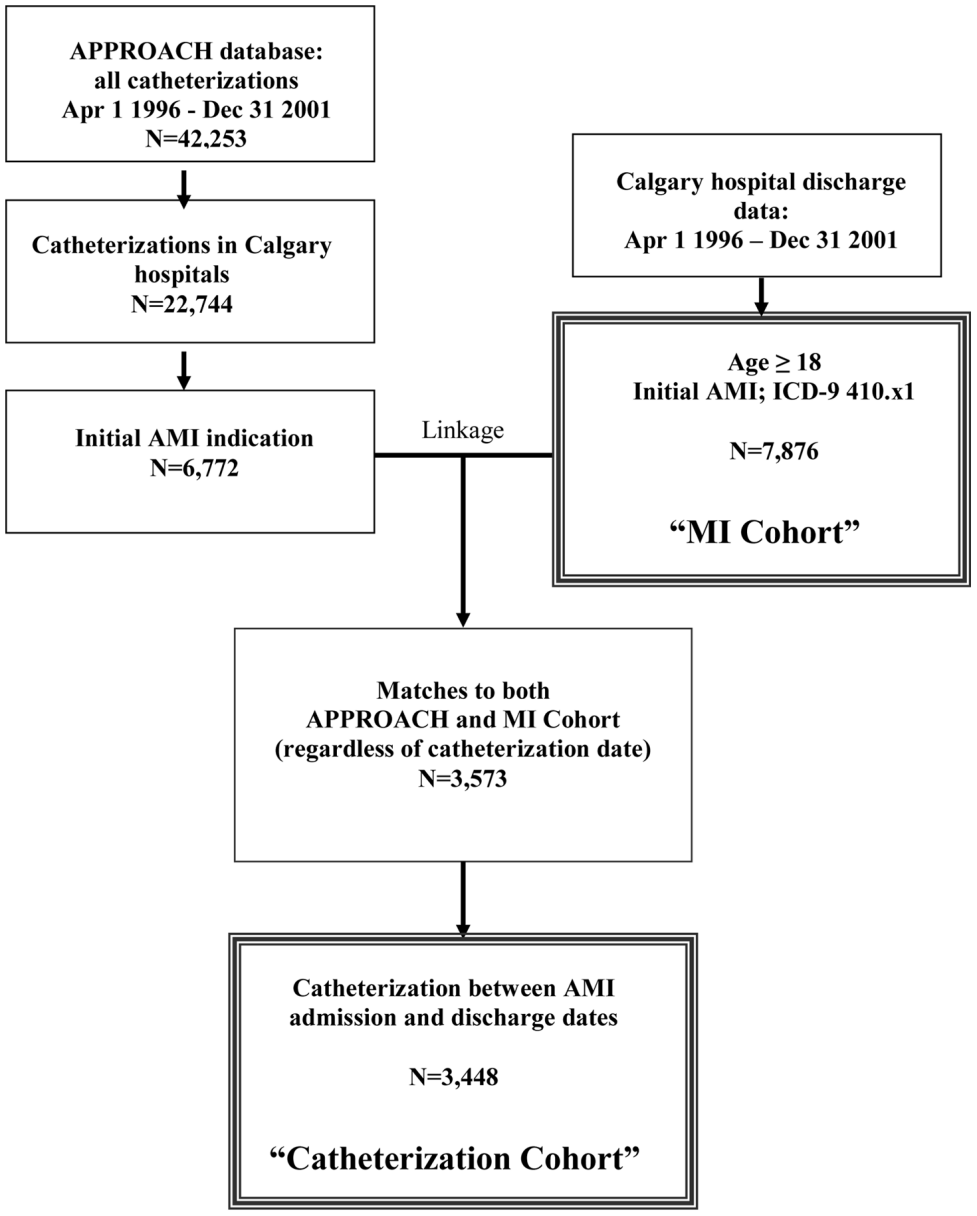
Flowchart of data sources.

Two data sources were used for this analysis (see Figure 1). First, we defined an MI Cohort using hospital discharge data from the Calgary hospitals that perform cardiac catheterization. All discharges between April 1, 1996 and December 31, 2001 for initial AMI (ICD-9-CM code 410.x1) were identified, and a patient's data were included in the MI Cohort if the patient was 18 years of age or older and lived in the Calgary Health Region. This earlier time period was studied because administrative data were coded in ICD-9-CM at that time and this verification of the International Classification of Diseases was needed for defining substance use disorders using a validated coding method (see details in next section). Only the first AMI admission between the specified dates was included for each patient. The MI Cohort data were used to define a subgroup with alcohol and/or drug use disorders (see below for details) as well as to provide information on in-hospital death, and on age, sex, and baseline patient comorbidities that could affect mortality and receipt of cardiovascular procedures. Calgary Health Region discharge data contain up to 16 ICD-9-CM diagnostic codes. When patients left hospital against medical advice, this was also captured by a code in the MI Cohort data.

Information on catheterization and revascularization procedures was obtained from APPROACH, a clinical data collection initiative capturing prospective information on all patients undergoing cardiac catheterization in Alberta, Canada since 1995 [21]. APPROACH contains detailed patient information including age, sex, ejection fraction, and the presence or absence of previous myocardial infarction (MI), congestive heart failure, diabetes, cerebrovascular disease, peripheral vascular disease, chronic pulmonary disease, elevated creatinine, dialysis, hyperlipidemia, hypertension, liver or gastrointestinal disease, or malignancy. It contains both historical and prospective information on therapeutic interventions such as thrombolytic therapy and revascularization by CABG or PCI. Coronary anatomy and procedural details are also recorded. Follow-up mortality is ascertained through semi-annual linkage to data from the Alberta Bureau of Vital Statistics. It has been shown that such detailed information is important in modelling revascularization rates, which cannot be adequately explained by baseline data available in discharge data alone [24].

To investigate revascularizations related to hospitalizations for initial AMI, the discharge data for all initial AMI admissions were linked to the APPROACH data to create a Catheterization Cohort, using the unique personal health number and the timing of catheterization (see Figure 1). Since we wished to assess revascularizations related to the current AMI, we only included linkages to catheterizations that occurred during the hospital admission [25].

The Ethics Review Board at the University of Calgary has approved the APPROACH protocol and the linkage of the APPROACH database with Calgary Health Region discharge data. Only data on patients who signed informed consent were included in the linkage.

### Definition of substance use disorders

Studies using administrative data have taken several different approaches to the definition of alcohol and drug use disorders using International Classification of Diseases, Ninth Revision, Clinical Modification (ICD-9-CM) codes. For example, Elixhauser's definitions focus on the psychiatric manifestations of abuse or dependence [26]. On the other hand, studies by both Adams and Saitz broadened the scope of the criteria to include medical sequelae of the substance use (that by definition indicate that the patient had the substance disorder) in the coding algorithms [21], [27]. A third algorithm has been proposed by the Washington Circle, a policy group on performance measurement for substance abuse services (www.washingtoncircle.org). They have defined a set of ICD-9-CM markers for addictions that is available on their website. This definition focuses on current alcohol or drug abuse, includes physical manifestations of abuse, and excludes substance abuse in remission codes.

Clinicians are more likely to under-report substance use disorders than to over-report them (these disorders are classically unrecognized and under-documented in records). Furthermore, the mechanism by which having a disorder might lead to worse care does not require the disorder be current (e.g. stigma from a past disorder may be relevant). Therefore we decided to use as sensitive a definition as possible, including physical manifestations of abuse such as alcoholic fatty liver. A list of candidate ICD-9-CM diagnostic codes was compiled by combining codes from 1) Drug and alcohol abuse as defined by Elixhauser and colleagues [26], 2) Drug- and alcohol-related diagnoses as defined by Saitz and colleagues [22] and 3) the Washington Circle definition (www.washingtoncircle.org), augmented by including substance use disorders in remission. This candidate list was then independently reviewed by two internists (RS and WG) and a psychiatrist (CB) to decide whether each ICD-9-CM code should be included in the definition, and to suggest other codes that might have been overlooked. Discrepancies were resolved by consensus.

Table 1 presents the resulting ICD-9-CM coding algorithm used to define substance use disorder (SUD) for the purpose of this analysis. A patient was considered to have an SUD if they had at least one of these codes in the discharge data associated with their AMI admission.

**Table 1:** Alcohol and other drug use disorder definition codes.

	Definition	ICD-9-CM
**Drug Use Disorders**	Opioid type drug dependence	304.0
	Barbiturate & similarly acting sedative or hypnotic drug dependence	304.1
	Cocaine drug dependence	304.2
	Cannabis drug dependence	304.3
	Amphetamine & other psycho stimulant drug dependence	304.4
	Hallucinogen drug dependence	304.5
	Other specified drug dependence	304.6
	Combinations of opioid type drug with any other	304.7
	Combinations of drug dependence excluding opioid type drug	304.8
	Unspecified drug dependence	304.9
	Cannabis abuse	305.2
	Hallucinogen abuse	305.3
	Barbiturate & similarly acting sedative or hypnotic abuse	305.4
	Opioid abuse	305.5
	Cocaine abuse	305.6
	Amphetamine or related acting sympathomimetic abuse	305.7
	Antidepressant type abuse	305.8
	Other mixed or unspecified drug abuse	305.9
	Drug withdrawal syndrome	292.0
	Hallucinogen poisoning	969.6
	Heroin poisoning	965.01
	Drug-induced dementia	292.82
	Drug-induced amnestic syndrome	292.83
	Drug-induced organic affective syndrome	292.84
	Other specified drug-induced mental disorders	292.89
	Unspecified drug-induced mental disorder	292.9
	Drug-induced organic delusional syndrome	292.11
	Drug-induced hallucinosis	292.12
	Pathological drug intoxication	292.2
	Drug dependence in the mother classifiable elsewhere, but complicating pregnancy, childbirth or the puerperium	648.3
**Alcohol Use Disorders**	Alcohol Abuse	305.0
	Acute alcoholic intoxication	303.0
	Other unspecified alcohol dependence	303.9
	Alcohol withdrawal delirium	291.0
	Alcohol amnestic syndrome	291.1
	Other alcoholic dementia	291.2
	Alcohol withdrawal hallucinosis	291.3
	Alcoholic jealousy	291.5
	Other specified alcoholic psychosis	291.8
	Unspecified alcoholic psychosis	291.9
	Alcoholic fatty liver	571.0
	Acute alcoholic hepatitis	571.1
	Alcoholic cirrhosis of liver	571.2
	Alcoholic liver damage, unspecified	571.3
	Alcoholic cardiomyopathy	425.5
	Alcoholic gastritis	535.3
	Alcoholic polyneuropathy	357.5
	Alcoholic pellagra	265.2
	Personal history of mental disorder alcoholism	V11.3
	Excessive blood level of alcohol	790.3
	Toxic effect of alcohol	980.0

### Statistical Analysis

#### Mortality, catheterization, and revascularization among all AMI admissions

The MI Cohort data were used to analyze in-hospital mortality and proportions receiving catheterization and revascularization among all patients admitted for initial AMI, regardless whether these individuals' data linked to the APPROACH database. Revascularization was studied as receipt of CABG, PCI, and “any revascularization” (ie. either CABG or PCI). For this analysis the catheterizations and revascularizations were restricted to within a year after the admission for AMI.

First, crude proportions for the SUD and no SUD groups were computed and compared for catheterization, revascularization, and in-hospital mortality using chi-square tests. Next, logistic regression was used to compare these outcomes between SUD groups adjusting for age, sex, and patient comorbidities. For mortality, we adjusted for comorbidities that have been shown by Tu and colleagues [28] to be predictive of mortality following acute MI. For receipt of catheterization and revascularization, we adjusted instead using Elixhauser's comorbidities [26], which are predictive of a more general group of outcomes for adult inpatients, including length of stay and costs as well as in-hospital mortality. To these Elixhauser comorbidities we added smoking, shock, and edema because these are particularly important for outcomes after AMI.

Since age might be expected to modify the effect of SUD on mortality and revascularization and past studies have tended to focus either on younger or older adults but not both, further logistic regression models were constructed to examine this potential interaction.

#### Revascularization following catheterization

Receipt of revascularization was examined in the Catheterization Cohort. First, we computed and compared the proportions receiving any revascularization (CABG or PCI) in each of the SUD groups using chi-square tests. Next, we examined time to revascularization using multivariable Cox proportional hazards analyses. Hazard ratios were calculated for revascularization at one year by SUD status, adjusting for age, sex, APPROACH comorbidities and severity of coronary artery disease. The proportional hazards assumption was tested using the method of Schoenfeld residuals. Further Cox models were constructed examining the interaction of age and SUD. Time-to-event curves were computed and plotted using a ‘cumulative incidence competing risks’ analysis, comparing the revascularization rates in the SUD and No SUD groups [29]. We chose this method over the more traditional Kaplan-Meier approach because it does not assume independence of death and revascularization.

#### Discharge against medical advice

It has previously been reported that outcomes can be misleading when discharge against medical advice (AMA) is not taken into account in studies of relationships between service utilization and substance use disorders [22], [23]. Therefore, we conducted a sensitivity analysis by excluding those individuals who left AMA, and repeating the analyses described above.

All analyses were conducted using SAS, Version 9.2 (Cary, NC) [30].

## Results

### Mortality, catheterization, and revascularization among all initial AMI admissions

#### Data sources and study population

There were 7,876 first admissions to Calgary Health Region hospitals for AMI between April 1, 1996 and December 31, 2001 (Figure 1). Table 2 presents the characteristics of these MI Cohort patients, of whom 203 (2.6%, 95%CI 2.2–3.0) met criteria for an SUD as defined in the Methods section. Of these, 174 (2.2%) had an alcohol use disorder, 44 (0.6%) had a drug use disorder, and 15 (0.2%) had both. Those with an SUD were more likely to be under age 65, to be male, to smoke, and to have certain related comorbidities (other neurological disorders, chronic pulmonary disease, liver disease, peptic ulcer disease, coagulopathy, fluid and electrolyte disorders, blood loss anemia, depression, and acute renal failure). Patients with SUDs were less likely to have cancer.

**Table 2:** Characteristics of SUD* and No SUD subgroups of the MI Cohort (N = 7,876).

		SUD* (N = 203)	No SUD*(N = 7,673)	p-value
**Age≥65**		74 (36.5%)	3,876 (50.5%)	<0.001
**Male**		170 (83.7%)	5,352 (69.8%)	<0.001
**Elixhauser comorbidities** **	**Congestive heart failure**	55 (27.1%)	1,676 (21.8%)	0.075
	**Cardiac arrhythmias**	31 (15.3%)	1,377 (18.0%)	0.326
	**Valvular disease**	22 (10.8%)	714 (9.3%)	0.459
	**Pulmonary circulation disorders**	5 (2.5%)	112 (1.5%)	0.243
	**Peripheral vascular disease**	15 (7.4%)	498 (6.5%)	0.609
	**Hypertension uncomplicated**	77 (37.9%)	2,941 (38.3%)	0.908
	**Hypertension complicated**	0 (0.0%)	9 (0.1%)	1.000†
	**Paralysis**	0 (0.0%)	40 (0.5%)	1.000†
	**Other neurological disorders**	17 (8.4%)	228 (3.0%)	<0.001
	**Chronic pulmonary disease**	36 (17.7%)	807 (10.5%)	0.001
	**Diabetes uncomplicated**	30 (14.8%)	1,255 (16.4%)	0.548
	**Diabetes complicated**	6 (3.0%)	258 (3.4%)	0.751
	**Hypothyroidism**	12 (5.9%)	505 (6.6%)	0.704
	**Renal failure**	12 (5.9%)	316 (4.1%)	0.207
	**Liver disease**	13 (6.4%)	14 (0.2%)	<0.001†
	**Peptic ulcer disease**	12 (5.9%)	219 (2.9%)	0.011
	**AIDS**	0 (0.0%)	0 (0.0%)	-
	**Lymphoma**	0 (0.0%)	17 (0.2%)	1.000†
	**Metastatic cancer**	0 (0.0%)	34 (0.4%)	1.000†
	**Solid tumor without metastasis**	5 (2.5%)	382 (5.0%)	0.102
	**Rheumatoid arthritis/collagen vascular diseases**	2 (1.0%)	147 (1.9%)	0.595
	**Coagulopathy**	11 (5.4%)	114 (1.5%)	<0.001†
	**Obesity**	11 (5.4%)	378 (4.9%)	0.741
	**Weight loss**	1 (0.5%)	13 (0.2%)	0.306†
	**Fluid & electrolyte disorders**	27 (13.3%)	484 (6.3%)	<0.001†
	**Blood loss anemia**	6 (3.0%)	43 (0.6%)	0.002
	**Deficiency anemias**	11 (5.4%)	404 (5.3%)	0.923
	**Psychoses**	4 (2.0%)	80 (1.0%)	0.171
	**Depression**	9 (4.4%)	137 (1.8%)	0.013†
**Tu** ***	**Shock**	7 (3.5%)	371 (4.8%)	0.362
	**Diabetes with complications**	7 (3.5%)	270 (3.5%)	0.957
	**Congestive Heart Failure**	53 (26.1%)	1,644 (21.4%)	0.109
	**Cancer**	0 (0.0%)	175 (2.3%)	0.025
	**Cerebrovascular disease**	8 (3.9%)	286 (3.7%)	0.874
	**Edema**	4 (2.0%)	64 (0.8%)	0.098
	**Acute renal failure**	17 (8.4%)	302 (3.9%)	0.002
	**Chronic renal failure**	13 (6.4%)	317 (4.1%)	0.111
	**Dysrhythmias**	48 (23.7%)	1,686 (22.0%)	0.570
**Smoking** ‡		41 (20.2%)	750 (9.8%)	<0.001

* ***Substance Use Disorder Defined By Diagnostic Codes As Described In The Methods.***

** ***Used In MI Cohort Analysis for Adjustment Of Revascularization Outcomes.***

*** ***Used In MI Cohort Analysis For Adjustment Of Mortality Outcomes.***

† ***Fisher Exact Tests Used To Calculate P-Value.***

‡ ***Smoking Defined By ICD-9-CM Code 305.1.***

#### In-hospital mortality

The in-hospital mortality for those with a known SUD (18 of 203; 8.9%) in the MI Cohort did not differ significantly (p = 0.344) from those without an SUD (547 of 7,673; 7.1%). However, Table 3 presents a different picture once age, sex, and comorbidities are included as covariates in a logistic regression model of in-hospital mortality as a function of SUD. Those with an SUD had significantly higher odds of in-hospital death than those without an SUD in the multivariable analysis (OR = 2.02, 95%C.I. (1.10, 3.69)).

**Table 3:** Odds Ratios for in-hospital mortality and catheterization by SUD* status (N = 7,876).

	Unadjusted OR** (95%CI)	Adjusted OR** (95% CI)
**Mortality**	1.27 (0.78, 2.07)	2.02 (1.10, 3.69)†
**Catheterization**	0.81 (0.61, 1.07)	0.75 (0.55, 1.01)‡

* ***Substance Use Disorder Defined By Diagnostic Codes As Described In The Methods.***

** ***Odds Ratio for Those with SUD Compared To Those Without.***

† ***Adjusted For Age And Sex, Tu Comorbidities (See*** ***Methods*** ***).***

‡ ***Adjusted For Age, Sex, Elixhauser Comorbidities (See*** ***Methods*** ***), Shock, Edema and Smoking.***

#### Catheterization

The proportion of those with SUD undergoing catheterization during hospitalization for AMI (107 of 203; 52.7%) did not differ significantly (p = 0.131) compared to the No SUD group (4,451 of 7,673; 58.0%). However, multivariable logistic regression results in Table 3 demonstrate a borderline significant odds ratio of 0.75 (95% CI 0.55–1.01) of catheterization among those with an SUD compared to those without an SUD, once adjusted for age, sex, smoking, comorbidities and shock. Of note, removal of smoking from the model had minimal effect on the results.

#### Revascularization

The MI Cohort received a total of 3845 revascularizations within 4 years of index hospitalization, including 900 coronary artery bypass grafting (CABG) and 2945 percutaneous coronary intervention (PCI) procedures; some patients received more than one revascularization. Having an SUD did not significantly affect the proportions of either CABG or PCI alone in the MI cohort, possibly because of small numbers receiving each intervention in the SUD group (data not shown). However, when CABG and PCI were combined, there was a near-significant difference (p = .05) in overall receipt of revascularization between the SUD groups, with 39.9% revascularized among those with SUD and 46.9% without SUD.

#### Interaction of age and SUD status

Age modified the effect of SUD on in-hospital mortality in a logistic regression model (p = .04); the effect of SUD was much greater in patients under age 65 than in the older group (a shift from an OR of 1.00 (ref) to 3.75 in younger patients than it was for patients over 65 (only a small shift from an OR of 3.51 with no SUD to 3.78)). There was no such effect modification for receipt of catheterization.

#### Revascularization following catheterization (Analysis using Catheterization Cohort)

During the period used to define the MI Cohort (April 1, 1996 to December 31, 2001), a total of 22,744 cardiac catheterizations were performed in Calgary hospitals and recorded in the APPROACH database. Of these, 6,772 catheterizations were done for an indication of initial hospital presentation with acute MI (ICD-9 code 410.x1). Linkage of the APPROACH database with the MI Cohort data yielded 3,448 records, with catheterization date falling within admission and discharge dates, (Catheterization Cohort) describing patients who underwent cardiac catheterization for an initial AMI indication during an admission for AMI. Out of these individuals, 73 (2.1%, 95% CI 1.7–2.7%) satisfied our criteria for SUD.

Table 4 presents the clinical and demographic characteristics of the 3,448 Catheterization Cohort patients by SUD status. As in the MI Cohort, patients with an SUD were more likely to be under age 65 and to smoke, and they were marginally more likely to have a malignancy or a liver or gastrointestinal disease. By contrast they did not differ from patients without an SUD by sex, comorbidities, or measures of risk based on coronary artery anatomy or ejection fraction.

**Table 4:** Characteristics SUD* and No SUD subgroups of the Catheterization Cohort (N = 3,448).

	SUD*(N = 73)	No SUD*(N = 3,375)	p-value
**Age≥65**	17 (23.3%)	1,476 (43.7%)	0.001
**Male**	58 (79.5%)	2,457 (72.8%)	0.203
**APPROACH clinical data** **			
**Hypertension**	34 (46.6%)	1,609 (47.7%)	0.856
**Hyperlipidemia**	35 (48.0%)	1,580 (46.8%)	0.845
**Diabetes**	10 (13.7%)	620 (18.4%)	0.308
**Congestive heart failure**	10 (13.7%)	598 (17.7%)	0.373
**Pulmonary disease**	5 (6.9%)	371 (11.0%)	0.262
**Prior thromblytic therapy**	14 (19.2%)	840 (24.9%)	0.265
**Prior PCI** ***	4 (5.5%)	293 (8.7%)	0.335
**Peripheral vascular disease**	6 (8.2%)	239 (7.1%)	0.707
**Cerebrovascular disease**	6 (8.2%)	202 (6.0%)	0.449†
**Prior CABG** ***	2 (2.7%)	163 (4.8%)	0.582†
**Liver/GI disease**	8 (11.0%)	174 (5.2%)	0.055†
**Malignancy**	0 (0.0%)	169 (5.0%)	0.049†
**Renal disease**	3 (4.1%)	105 (3.1%)	0.498†
**Dialysis**	1 (1.4%)	39 (1.2%)	0.577†
**Smoking**			<0.001
**Previous smoker**	14 (19.2%)	997 (29.5%)	
**Current smoker** ‡	47 (64.4%)	1,152 (34.1%)	
**Anatomy**			0.823†
**Normal & <50%**	4 (5.5%)	130 (3.9%)	
**Low risk** ††	40 (54.8%)	1,825 (54.1%)	
**High risk** ‡‡	24 (32.9%)	1,192 (35.3%)	
**Left main**	5 (6.9%)	222 (6.6%)	
**Not Entered/missing**	0 (0.0%)	6 (0.2%)	
**Ejection Fraction**			0.396†
**>50**	45 (61.6%)	2,051 (60.8%)	
**35–50**	13 (17.8%)	848 (25.1%)	
**20–34**	6 (8.2%)	157 (4.7%)	
**<20**	0 (0.0%)	11 (0.3%)	
**Couldn't be done**	1 (1.4%)	18 (0.5%)	
**Not done**	7 (9.6%)	209 (6.2%)	
**Not entered/missing**	1 (1.4%)	81 (2.4%)	

* ***Substance Use Disorder Defined By Consensus as In Methods.***

** ***Methods of Data Collection Described In Ghali et.al.*** [***21***] ***.***

*** ***CABG = coronary artery bypass graft PCI = percutaneous coronary intervention.***

† ***Fisher Exact Tests Used To Calculate P-Value.***

‡ ***Current Smoker Includes Those Who Reported Quitting Less Than 3 Months Prior To Catheterization.***

†† ***Low Risk includes 1-Vessel Disease (VD) 50–75%, 1VD 95%, 2VD, 2VD both 95%, 1VD 95% Proximal Left Anterior Descending (PLAD), 2VD 95% Left Anterior Descending (LAD).***

‡‡ ***High Risk includes 2VD 95% PLAD, 3VD, 3VD 1–95%, 3VD PLAD, 3VD 95% PLAD.***

**Figure 2:**
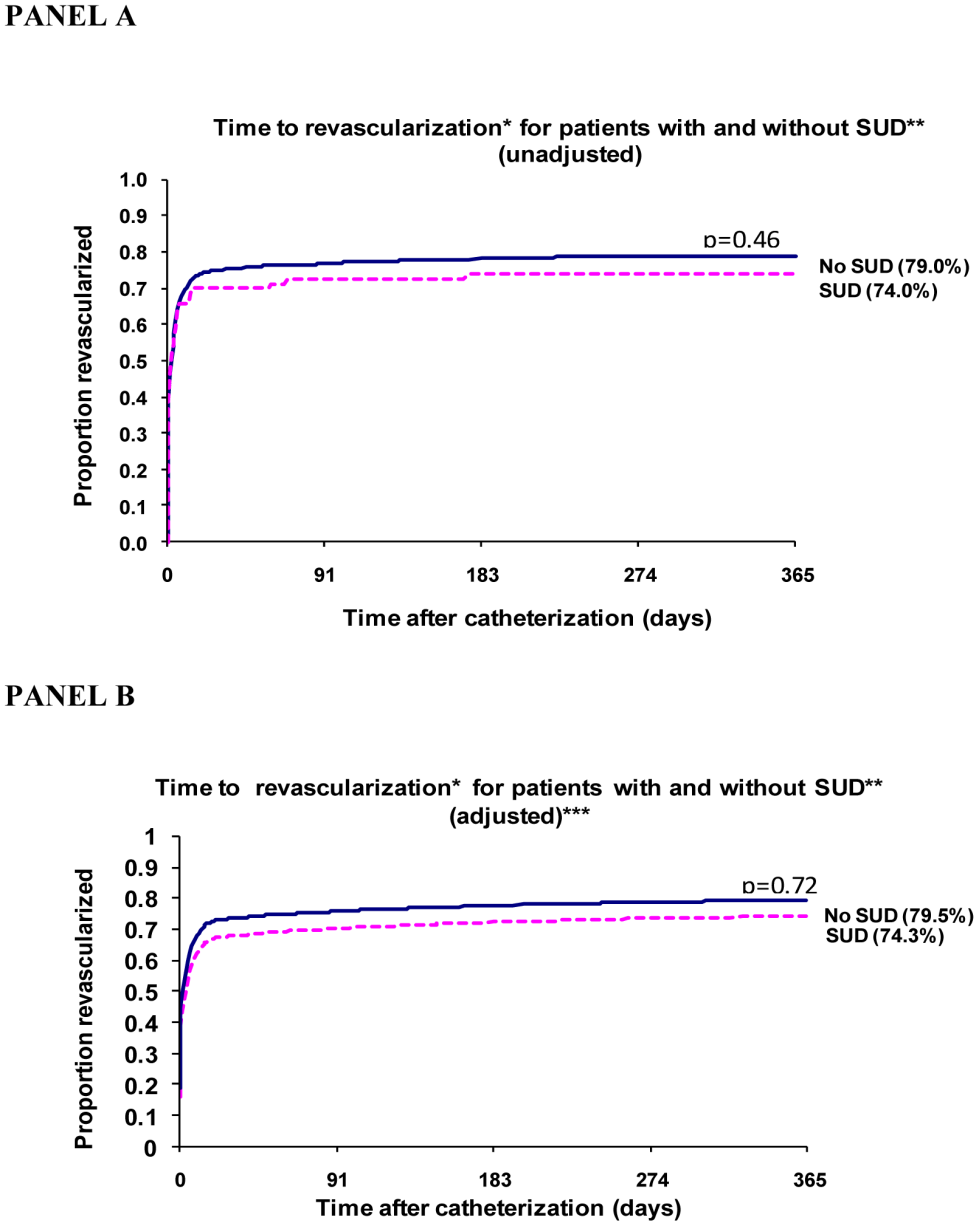
Time to Revascularization Curves (N = 3,448): Unadjusted (PANEL A) and Adjusted (PANEL B).

The time-to-event curves describing revascularization events as a function of time since catheterization for those with and without an SUD are shown in Figure 2. Within one year of catheterization, (2,717 of 3,375; 81%) of those without an SUD had undergone revascularization, compared with (55 of 73; 76%) with an SUD (p = 0.32). The unadjusted hazard ratio for any revascularization by one year post-MI was 0.91 (95% CI: 0.69 to 1.20) for those with an SUD compared to those without (Table 5). Adjusting for age, sex, severity of coronary artery disease (anatomy and ejection fraction), and comorbidities, the hazard ratio was 0.83 (95% CI: 0.44 to 1.50) (Table 5). The Schoenfeld residual test did not indicate violation of the proportional hazards assumption (p = 0.421).

**Table 5:** Hazard Ratios for CABG* and PCI** within one year by SUD† (N = 3,448).

	Unadjusted HR‡ (95% CI)	Adjusted†† HR‡ (95% CI)
**CABG** *	0.83 (0.47, 1.47)	0.63 (0.20, 1.98)
**PCI** **	0.97 (0.72, 1.30)	0.84 (0.42, 1.69)
**Any revascularization**	0.91 (0.69, 1.20)	0.83 (0.44, 1.50)

* ***Coronary Artery Bypass Grafting.***

** ***Percutaneous Coronary Intervention.***

† ***Substance Use Disorder Defined By Consensus as In Methods.***

‡ ***Hazard Ratio.***

†† ***Adjusted For Age, Sex, APPROACH Comorbidities, Smoking and Severity of CAD.***

#### Sensitivity analysis excluding patients discharged against medical advice

The results for receipt of catheterization differed in interpretation when the cohort was restricted to those who did not leave AMA, in that the adjusted odds of catheterization was significantly lower for the SUD group compared to No SUD (OR 0.72, 95% CI 0.53 to 0.97). In the full MI Cohort (including those who left AMA) this odds ratio had only approached significance (OR 0.75, 95% CI 0.55 to 1.01; see Table 3). For the revascularization and mortality endpoints, the results were essentially unchanged when patients who left AMA were excluded from the analysis (data not shown).

## Discussion

Our results suggest that alcohol and drug disorders are associated with a significantly higher odds of in-hospital mortality following AMI, and may be associated with decreased access to catheterization and subsequent revascularization. These results agree with some past reports from the United States, including those of Druss and Young [11], [12], and suggest that the Canadian universal health care environment does not eliminate disparities in access to cardiac procedures after AMI for those with substance use disorders. Moreover, our study extends previous results by clarifying that the association of substance use with access to catheterization is similar in younger and older adults. To our knowledge, our study is the first to examine these issues in adults of all ages taking into account clinical characteristics such as ejection fraction that affect decision making regarding cardiac procedures.

This study further demonstrates the importance of including individuals who leave hospital AMA in analyses of this type. Our catheterization results showed a significant discrepancy in receipt of catheterization post-AMI when the sample was restricted to those who did not leave AMA; this result was no longer significant in the full cohort including those who left AMA. The availability of an external database containing catheterization data allowed us to analyze these individuals' outcomes despite their leaving hospital.

In spite of our broad definition of drug and alcohol disorders, the sensitivity of our measure was probably low, as evidenced by the prevalence of drug or alcohol disorders of only 2.3 per cent. In our study, this misclassification would be expected to produce a conservative result, as several individuals with substance disorders would be included in the non-substance use disorder group. Juergens and colleagues have pointed out a similar lack of sensitivity of physician recorded diagnoses of alcoholism in the coronary care unit, and showed that it can be mitigated by screening [31]. Furthermore, our patient numbers were sufficient to demonstrate a discrepancy in receipt of catheterization post-AMI and provides insight into potential areas for intervention.

Another limitation in our study is that we are unable to distinguish between ST-elevation and non ST-elevation MI. The importance of this limitation is mitigated by the fact that early catheterization has been shown to be of value in both sub-types of MI [32]. We recognize that the data studied is from a time when standard of care may have been different. This was done as we used validated ICD-9-CM definitions for SAD's, and therefore we have confined the analysis to older data. We do not think however that it undermines the internal validity of the comparison between those with substance abuse vs. those without.

The reason for increased mortality in the SUD group may relate partially to lower quality of care, though we cannot be certain based on our data alone. It is also possible that we inadequately adjusted for smoking by relying on ICD-9 codes rather than clinical report in the analysis of the MI cohort. A final caveat is that although we adjusted for major comorbidities and clinical characteristics, it is possible that there are other variables for which we did not adjust that might have influenced the outcome.

The reason for the decreased access to cardiac procedures seen in our data and in that of other groups is as yet unknown. There is known variability unrelated to patient characteristics in ratings of appropriateness of treatment [33]. Whether issues of stigma might enter into the decision-making process regarding cardiac procedures for individuals with substance use disorders is unclear at this point, though there is some literature to suggest that stigma does limit access to other types of medical care for these individuals [34], [35].

In interpreting these results, it is also important to recognize that patients do not always consent to procedures which are offered to them, and that refusal of procedures was not available as a variable in our data. It is possible that individuals with substance use disorders in our sample were more likely to refuse procedures than those without such disorders. This would be consistent with the literature that reports an association of drug and alcohol disorders with leaving hospital against medical advice [22], [36], [37]. Although we had similar results when we excluded individuals who signed out against medical advice, there might have been others who refused a catheterization or revascularization, but were not discharged against medical advice.

In conclusion, we demonstrate that despite a universal health care system, individuals post AMI both under and over the age of 65 with alcohol and drug use disorders have a higher likelihood of in-hospital mortality, and a lower likelihood of receiving cardiac procedures, than those without substance use disorders. Our finding of higher mortality in the face of this poorer access to procedures suggests that individuals with drug and alcohol use disorders might be undertreated following AMI. Targeted efforts are required to explore the interplay of patient and provider factors that underlie this finding so that subsequent interventions can be developed to ultimately enhance the quality of care. Qualitative investigation might be a next step to better define the underlying barriers, beliefs, and processes that are operating.
